# Tuning oxygen impurities and microstructure of nanocrystalline silicon photovoltaic materials through hydrogen dilution

**DOI:** 10.1186/1556-276X-9-303

**Published:** 2014-06-14

**Authors:** Chao Wen, Hao Xu, Wei He, Zhengping Li, Wenzhong Shen

**Affiliations:** 1 Key Laboratory of Artificial Structures and Quantum Control (Ministry of Education), Institute of Solar Energy, Department of Physics, Shanghai Jiao Tong University, Shanghai 200240, People's Republic of China

**Keywords:** Nanocrystalline silicon, Hydrogen dilution, Oxygen impurities, Bonded hydrogen, Grain boundaries

## Abstract

As a great promising material for third-generation thin-film photovoltaic cells, hydrogenated nanocrystalline silicon (nc-Si:H) thin films have a complex mixed-phase structure, which determines its defectful nature and easy residing of oxygen impurities. We have performed a detailed investigation on the microstructure properties and oxygen impurities in the nc-Si:H thin films prepared under different hydrogen dilution ratio treatment by the plasma-enhanced chemical vapor deposition (PECVD) process. X-ray diffraction, transmission electron microscopy, Raman spectroscopy, and optical transmission spectroscopy have been utilized to fully characterize the microstructure properties of the nc-Si:H films. The oxygen and hydrogen contents have been obtained from infrared absorption spectroscopy. And the configuration state of oxygen impurities on the surface of the films has been confirmed by X-ray photoelectron spectroscopy, indicating that the films were well oxidized in the form of SiO_2_. The correlation between the hydrogen content and the volume fraction of grain boundaries derived from the Raman measurements shows that the majority of the incorporated hydrogen is localized inside the grain boundaries. Furthermore, with the detailed information on the bonding configurations acquired from the infrared absorption spectroscopy, a full explanation has been provided for the mechanism of the varying microstructure evolution and oxygen impurities based on the two models of ion bombardment effect and hydrogen-induced annealing effect.

## Background

As a low-cost, high-efficiency thin-film material, hydrogenated nanocrystalline silicon (nc-Si:H) has emerged as a very attractive candidate for the application of next-generation solar cells. Extensive optical and electrical investigations have been carried out to reveal the favorable features of nc-Si:H thin films such as tunable bandgap, strong optical absorption, high carrier mobility, and great stability against light soaking [1-4]. High efficiency and good stability of single-junction [5] and tandem [6,7] third-generation nc-Si:H thin-film solar cells have been realized based on the good properties of this material. The initial active-area efficiency of a triple-junction structured cell has been demonstrated to be 16.3% [8] by taking advantage of the nc-Si:H material.

However, the nc-Si:H film is in nature a mixed-phase structure consisting of nanometer-sized grains embedded in an amorphous matrix [9], which determines that the defect microstructures such as grain boundaries and voids exist in the films with a large volume fraction. And oxygen impurities from post-deposition oxidation can easily diffuse into the film because of the porous defect structure and induce additional defects [10] within the nc-Si:H films as well. Furthermore, incorporation of oxygen into the nc-Si:H films can lower the optical absorption [11] of amorphous Si (a-Si)-based solar cells when nc-Si:H films are used as a window layer or tunnel junction [12]. It has also been found that nc-Si:H is more sensitive to oxygen impurities than a-Si:H because oxygen can form weak donors in nc-Si:H materials, which raises the Fermi level towards the conduction band [13]. Therefore, it is of significant importance to regulate the defect structure and the oxygen impurities in the films in order to better the performance of nc-Si:H material-based solar cells.

In this work, we have performed a detailed structural and optical investigation on nc-Si:H thin films with hydrogen dilution profiling to analyze the structure evolution and oxygen incorporation under the influence of hydrogen. The bonding configuration of surface oxygen has been identified by the X-ray photoelectron spectroscopy (XPS) spectra. Moreover, a detailed analysis on the infrared Si-H stretching mode has been given to reveal the tuning mechanism of hydrogen on structure and oxygen impurities during the growth process based on the two models of ion bombardment effect and hydrogen-induced annealing effect.

## Methods

The nc-Si:H thin films were grown on both glass and double-side-polished intrinsic single-crystalline silicon (c-Si) (100) substrates by a capacitively coupled plasma-enhanced chemical vapor deposition (PECVD) system with the gases SiH_4_ and H_2_. The PECVD system was operated at a radiofrequency (RF) of 13.56 MHz, an RF power density of 0.4 W/cm^2^, a total gas flow rate of 120 sccm, a chamber pressure of 150 Pa, and a temperature of 250°C. The hydrogen dilution ratio *R*_H_ [H_2_/(H_2_ + SiH_4_)] varied from 97.5% to 99.2%. The detailed physical characteristics of the nc-Si:H samples are summarized in Table 1.

**Table 1:** Summary of physical parameters of the nc-Si:H thin films prepared under various hydrogen dilution ratios

***R***_**H **_**(%)**	***R***_**d **_**(Å/s)**	***d *** **(nm)**	***X***_**C **_**(%)**	***n***_**∞**_	***C***_**O **_**(at.%)**	***C***_**H **_**(at.%)**
97.5	0.2895	8.6	76.83	2.980	5.73	34.19
98.0	0.2583	7.3	75.41	2.768	8.39	33.90
98.2	0.2540	6.3	73.15	2.744	8.80	32.46
98.6	0.1966	5.8	72.07	2.663	10.92	33.98
98.8	0.1830	5.5	74.69	2.650	9.34	33.66
99.2	0.1778	6.1	75.72	2.541	3.33	30.63

The film thicknesses were directly measured by a Dektak 6 M profilometer (Veeco Instruments Inc., Plainview, NY, USA). The average grain size *d* was derived from the (111) X-ray diffraction (XRD) peak, measured with a Bruker D-8 XRD system (Cu Kα radiation, 40 kV and 60 mA, Madison, WI, USA) at room temperature, and the grain size was also directly observed by high-resolution transmission electron microscopy (HRTEM; CM200, Philips, Amsterdam, The Netherlands). The crystalline volume fraction *X*_C_ was calculated from the Raman spectra, measured with a Jobin Yvon LabRam HR800 UV micro-Raman spectrometer (backscattering configuration and Ar ion laser of 514.5 nm, Kyoto, Japan). The laser power density is 1 mW/mm^2^ to avoid any beam-induced crystallization. The long-wavelength limit of the refractive index *n*_∞_ was deduced from optical transmission spectra, measured with the double-beam ultraviolet-visible-near-infrared spectrometer PerkinElmer UV Lambda 35 (300- to 1,000-nm spectral range with 0.5-nm resolution, Waltham, MA, USA).

The hydrogen (oxygen) content bonded to silicon *C*_H_ (*C*_O_), and its bonding configurations were obtained from infrared (IR) absorption spectra, measured with a Nicolet Nexus 870 Fourier transform IR spectrometer (400 to 4,000 cm^-1,^ Thermo Fisher Scientific Inc., Waltham, MA, USA). XPS was used to study the silicon core energy level of the nc-Si:H. All the spectra were obtained with an electron takeoff angle of 90° using an Al Kα source monochromatic X-ray radiation. The Kratos charge neutralizer system (Kratos Analytical, Chestnut Ridge, NY, USA) was used on all the samples to compensate the charging effect of the sample surface. The narrow scan of the spectra was collected at a high-resolution mode with a pass energy of 20 eV. The binding energy was calibrated to the C1s emission (284.8 eV) arising from surface contamination. The background from each spectrum was subtracted using a Shirley-type background to remove most of the extrinsic loss structure. All the comparative data and spectra presented below are normalized with thickness.

## Results and discussion

**Figure 1:**
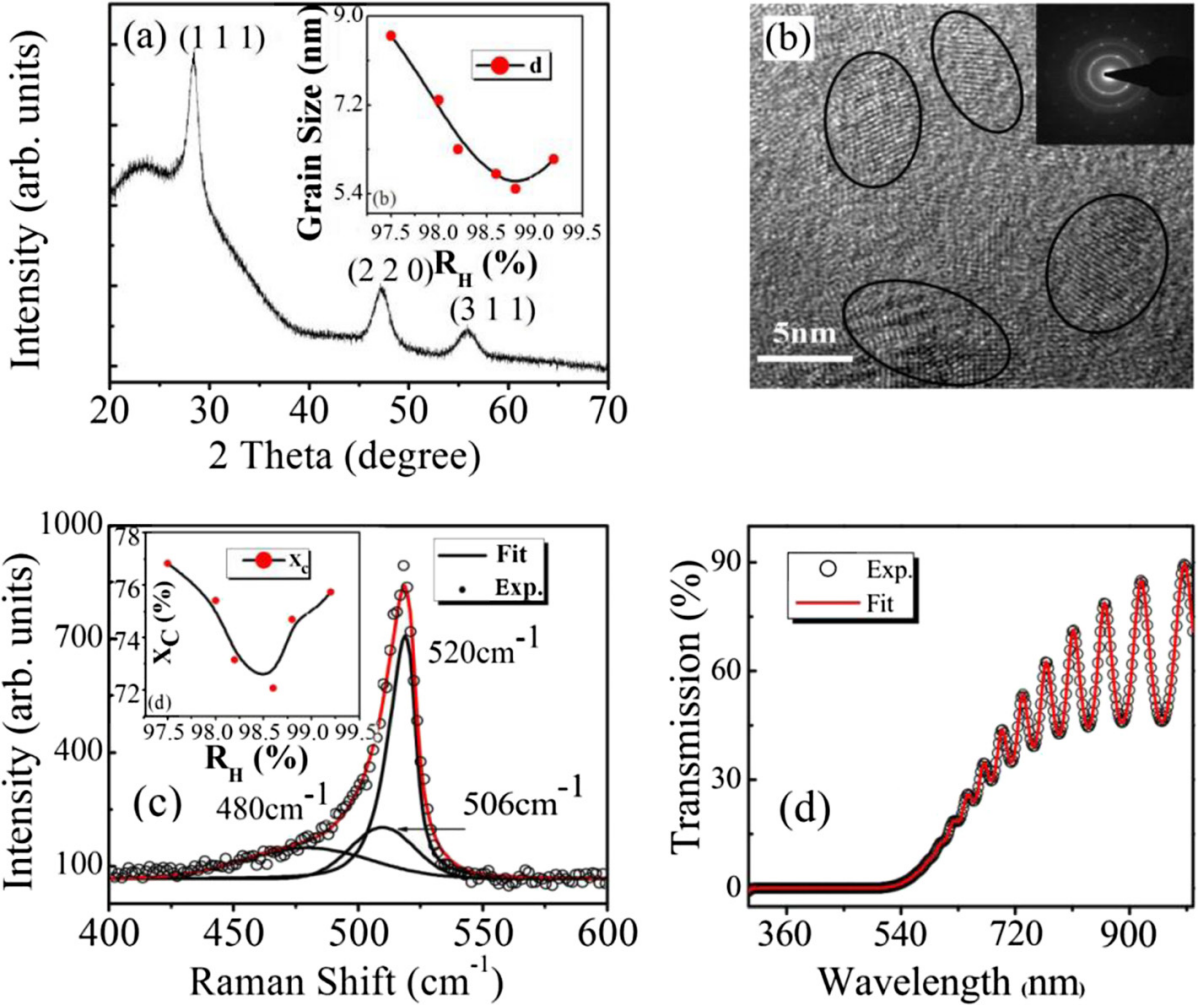
**Structural and optical properties of a representative nc-Si:H sample with***R*_H_ **= 98.2%. (a)** Experimental XRD spectrum showing diffraction peaks (111), (220), and (311). The inset shows the average grain sizes of the films under different *R*_H_. **(b)** The image of HRTEM with an inset of the SAED pattern. **(c)** Experimental (open circles) and fitted (solid curve) Raman spectrum with the inset presenting the crystalline volume fractions within the films under different *R*_H_. **(d)** Experimental (open circles) and fitted (solid curve) optical transmission spectrum.

To investigate the structural properties of the nc-Si:H thin films grown under various H dilution profiling, micro-Raman and XRD measurements were carried out. In Figure 1a, the XRD pattern for the sample with *R*_H_ = 98.2% is presented, in which the three diffraction peaks appearing at 2*θ* ~ 29.0°, 47.5°, and 57.0° correspond to the (111), (220), and (311) planes of c-Si, respectively. The presence of large diffraction peak broadening of (111), (220), and (311) c-Si peaks indicates the appearance of a silicon nanocrystalline phase in the film. The strongest XRD peak intensity for the (111) plane indicates that the nanocrystallites have preferentially grown along the (111) direction. Based on the Scherrer formula [14], the average grain size *d* in the (111) direction was calculated to be approximately 5.8 nm, which is in good agreement with the value directly observed from HRTEM as shown in Figure 1b. The provided selected area electron diffraction (SAED) pattern in the inset of Figure 1b shows the diffraction rings of the (111), (220), and (311) planes of silicon, which further ascertains the two-phase-mixture nature of the nc-Si:H thin films. It can be clearly observed from the inset of Figure 1a that with the increase of *R*_H_ up to 98.8%, the grain size *d* has a significant decrease from the maximum value of 8.6 to 5.5 nm in the nc-Si:H thin films. And further increasing the hydrogen dilution to 99.2% only leads to a slight increment of *d*. As we will discuss below, this can be, in principle, due to the depletion of deposited SiH_*x*_ radical molecules by the hydrogen flux.

Figure 1c shows the typical experimental result of Raman spectrum corresponding to the sample with *R*_H_ = 98.2%. The spectrum was decomposed into three satellite spectra, namely a broad Gaussian distribution around 480 cm^-1^ resulting from the transverse optical (TO_1_) mode of amorphous silicon, a Lorentzian peak near 520 cm^-1^ coming from the asymmetric TO_2_ vibrational mode of crystalline silicon [15], and one peak around 506 cm^-1^ originating from the intermediate mode of crystal-like phase at grain boundaries [16]. The crystalline volume fraction *X*_C_ of the nc-Si:H films can be estimated from the relation *X*_C_ = (*I*_A_ + *I*_GB_)/(*I*_C_ + *I*_GB_ + *I*_A_), where *I*_A_, *I*_GB_, and *I*_C_ are the integrated peak intensity at 480, 506, and 520 cm^-1^, respectively. And the obtained crystalline volume fraction *X*_C_ vs. hydrogen dilution ratio *R*_H_ was plotted in the inset of Figure 1c. According to both the surface model [17] and the growth zone model [18], increasing *R*_H_ will result in an increase of *X*_C_. However, our experimental results show that *X*_C_ increases only when *R*_H_ is higher than 98.8%, and hence, the decrease of *X*_C_ in the *R*_H_ range up to 98.6% cannot be fully explained by the mentioned growth models. Therefore, additional discussion involving the hydrogen ion bombardment [19] effect is necessary to fully explain the film growth mechanism as well as to understand the structure characterization.

Optical transmission measurements were performed at room temperature to generate optical information on the nc-Si:H thin-film samples. Figure 1d displays the experimental (open circles) and fitted (solid curve) optical transmission spectrum for the sample with *R*_H_ = 98.2%. From the fitting process conducted within the framework of a modified four-layer-medium transmission model based on the envelop method [20], both the film thickness (approximately 636.9 nm) and the long-wavelength limit of the refractive index (*n*_∞_ ~ 2.663) were obtained. The thicknesses of the films are in good agreement with the values directly measured by the step profilometer as listed in Table 1. And the long-wavelength limit of the refractive index *n*_∞_ is an important optical parameter associated with the mass density and atomic structure of nc-Si:H thin films, which together with the *X*_C_ obtained from the Raman measurement can be used to calculate the respective volume fractions of the three components, namely c-Si, a-Si, and voids in the films. Table 1 summarizes the structural and optical properties of the nc-Si:H thin films under various *R*_H_.

**Figure 2:**
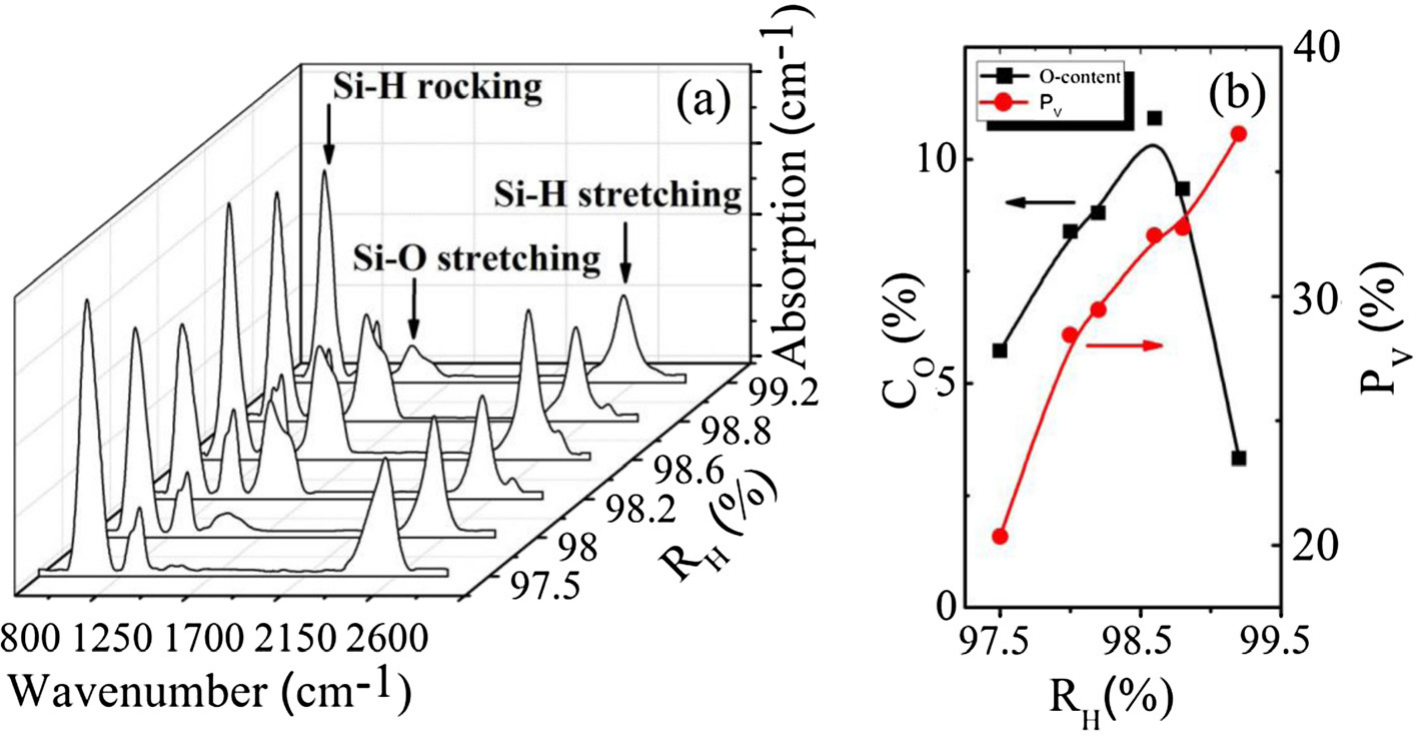
**IR absorption spectra and oxygen content and volume fraction of voids. (a)** IR absorption spectra of the nc-Si:H thin films prepared under different *R*_H_. **(b)** Oxygen content and volume fraction of voids as a function of *R*_H_.

Finally, room-temperature IR transmission measurements were conducted to obtain both the oxygen content and hydrogen content in these films. Figure 2a shows the IR absorption spectra of the samples prepared under different *R*_H_, with four major absorption peaks appearing at around 630 cm^-1^ (Si-H rocking-wagging mode), 880 cm^-1^ (Si-H bending mode), 1,030 cm^-1^ (Si-O stretching mode), and 2,090 cm^-1^ (Si-H stretching mode) [21-24]. In the calculation of the absorption coefficient, the transmittance was normalized to eliminate the interference fringes due to the small index of refraction difference between the c-Si substrate and the films. The bonded oxygen content *C*_O_ can be yielded by numerical integration of the peak around 1,000 to 1,200 cm^-1^, which is related to the Si-O-Si stretching mode through the equation *C*_O_ (at.%) = 1/*N*_Si_ × *A*_W_ × ∫(*α*(*ν*)/*ν*)*dν*, where *α*(*υ*) is the absorption coefficient of the film at wavenumber *υ*, *N*_Si_ = 5 × 10^22^ cm^-3^ is the atomic density of pure silicon, and the proportionality constant *A*_W_ is fixed to be 2.8 × 10^19^ cm^-2^[22,23]. The bonded hydrogen content *C*_H_ can also be calculated from the Si-H rocking mode at around 630 cm^-1^ with *A*_W_ = 2.1 × 10^19^ cm^-2^[25]. The calculated *C*_O_ and *C*_H_ for all these nc-Si:H films are listed in Table 1.

As a mixed-phase material with nanocrystallites embedded in an amorphous matrix, nc-Si:H contains a certain volume fraction of nanometer-sized voids, which should not be neglected when characterizing the microstructure of the films [26]. The volume fraction of voids *P*_V_ in these nc-Si:H thin films was calculated based on Bruggeman's effective media approximation [27] using the crystalline fraction *X*_C_ from the Raman analysis and the refractive index *n*_∞_ from the transmission calculation. As shown in Figure 2b, *P*_V_ increases monotonically with the increase of *R*_H_, which indicates that the presence of microvoids in these thin films is enhanced by hydrogen dilution and that the compactness of the samples decreases with the increase of *R*_H_.

As also shown in Figure 2b, the total oxygen content *C*_O_ for the samples initially has an increase from 3.33% to 10.92% with the increase of *R*_H_ up to 98.6%, and then a downshift of *C*_O_ occurs when further increasing *R*_H_. Researchers have found that most of the oxygen atoms were incorporated into the films through post-oxidation [28]. Concerning the material structure, cavities and voids in the material are probably crucial for accommodation of oxygen molecules. Hence, the variation of *C*_O_ along *R*_H_ is expected to be similar to that of *P*_V_. Nevertheless, our experimental data show an interesting nonmonotonic correlation that higher *P*_V_ is associated with less oxygen impurities when *R*_H_ is above 98.6%, which deviates from the above expectation. And the deviation indicates that there should be some other type of defect structure overwhelmingly affecting the incorporation of the oxygen inside the films rather than voids.

To fully understand the relation between the defect microstructure and the oxidation effects, it is quite necessary to investigate the structure evolution mechanism and to elucidate the hydrogen behavior in the growth process of the nc-Si:H thin film, which is a complex synergy between surface and bulk reactions of impinging SiH_*x*_.

**Figure 3:**
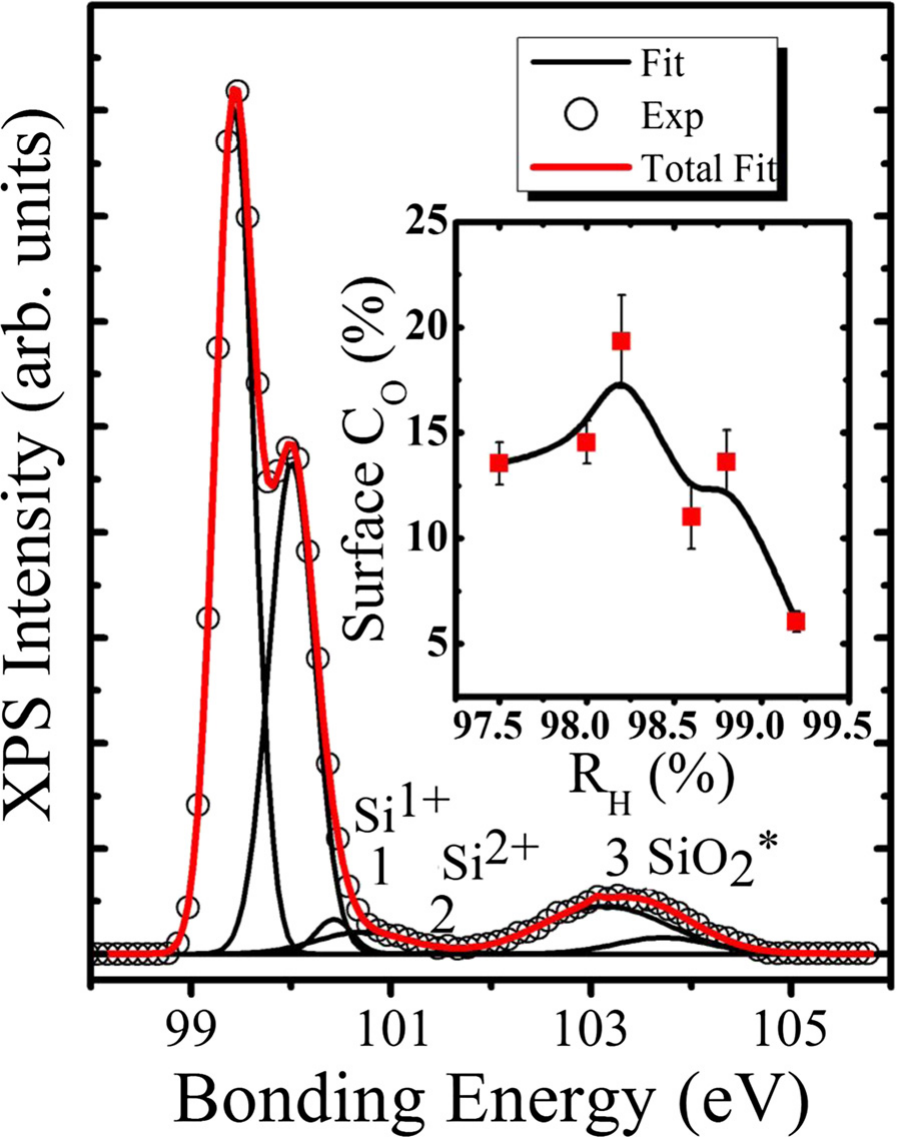
**Typical XPS Si 2p spectrum of the nc-Si:H thin film under***R*_H_ **= 98.2%.** The splitting of 0.6 eV is shown with all the intermediate oxidation states. The inset presents the surface oxygen content as a function of *R*_H_.

XPS measurements have been further employed to accurately investigate the Si/O surface interaction. Figure 3 displays a representative high-resolution Si 2p spectrum (from the sample with *R*_H_ = 98.2%) to understand the suboxide on the film surface. The synchrotron work of Himpsel et al. [29] and Niwano et al. [30] afforded the information for all energy level fitting. The fitting components generated from the decomposition of the measured spectrum correspond to different Si bonding states. For the as-fabricated nc-Si:H materials, the Si 2p region has been routinely fitted to Si 2p_1/2_ and Si 2p_3/2_ partner lines for Si^4+^, Si^0^, and intermediate states such as Si^1+^ (Si_2_O), Si^2+^ (SiO), and Si^3+^ (Si_2_O_3_). The additional component of silicon oxide was referred as SiO_2_*, which is assigned to be the regular crystalline-like phase produced at the interface of SiO_2_-Si. This part mainly comes from the lattice mismatch of the oxide and single-crystal Si^29^ with its peak located at a binding energy of 0.35 eV, slightly lower than that of SiO_2_. It can be confirmed from the above data analysis that Si^3+^ does not exist in the sample, while the existence of Si^1+^ and Si^2+^ species are supported by the XPS observation.

Moreover, we can notice from peak 3 that the nc-Si:H surface was well passivated with SiO_2_. Surface oxygen content as a function of *R*_H_ is presented in the inset of Figure 3. From the inset, we notice that the sample with *R*_H_ = 99.2% has a very low value of surface oxygen content, demonstrating that high *R*_H_ hydrogen effectively limits the intermediate oxide formation by passivation at the near surface. It can be clearly seen from the evolution of the surface oxygen content that the surface oxygen content first shifts towards the highest value of 24.32% upon increasing *R*_H_ up to 98.2%. But when *R*_H_ is further increased to 99.2%, the surface oxygen content downshifts towards the lowest value of 13.56%. Besides, *R*_H_ = 98.2% gives rise to the highest peak intensity of surface oxygen content while the oxygen content *C*_O_ in the bulk is not the highest. This may be related to the surface smoothness at the atomic level of the sample, i.e., a rough surface of the silicon material produces more intermediate oxidation states. The oxygen content *C*_O_ in the bulk is mainly influenced by H and the H-related defect structure, which we will discuss in the following part.

**Figure 4:**
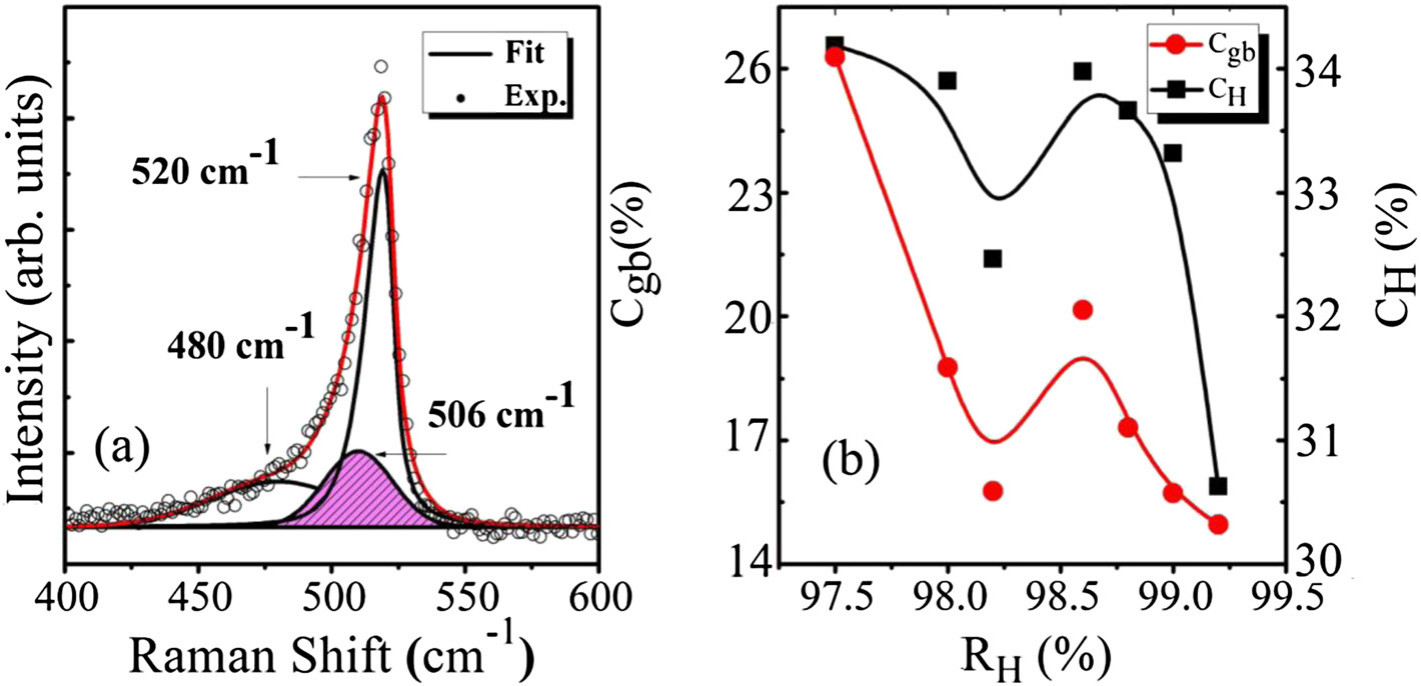
**Experimental and fitted Raman spectrum and volume fraction of grain boundaries and hydrogen content. (a)** Experimental (open circles) and fitted (solid curve) Raman spectrum of a representative sample with *R*_H_ **=** 98.2%. **(b)** Volume fraction of grain boundaries and hydrogen content as a function of *R*_H_.

As we mentioned in Figure 2b, there is a deviation between the oxygen impurities and the volume fraction of voids *P*_V_ when *R*_H_ is above 98.6%, which probably resulted from another important defect structure, that is, grain boundaries between the nanocrystallites and the amorphous matrix of the nc-Si:H films. We can get the information on grain boundaries from the Raman measurement. The Raman spectra of the nc-Si:H films were collected between 400 and 600 cm^-1^ using a confocal microscope with a laser having an excitation wavelength of 514 nm. The spectrum of a representative sample with *R*_H_ = 98.2% is shown in Figure 4a, which was deconvoluted into three component peaks at 520, 480, and 506 cm^-1^. These three deconvoluted peaks indicate the presence of well-ordered, disordered, and quasiordered silicon phases, respectively. The last peak has been taken by several authors to indicate the presence of grain boundaries [16], whose volume fraction (*C*_GB_) in nc-Si:H films can be estimated from the relation *C*_GB_ = *I*_GB_/(*I*_C_ + *I*_GB_ + *I*_A_), where *I*_A_, *I*_GB_, and *I*_C_ are the integrated intensities of the peaks observed at 480, 506, and 520 cm^-1^, respectively.

We show in Figure 4b the variation of *C*_GB_ and *C*_H_ as a function of *R*_H_. It can be clearly observed that *C*_GB_ and *C*_H_ have the same variation behavior as a function of *R*_H_, demonstrating that as an important defect microstructure, the volume fraction of grain boundaries in the nc-Si:H films can be effectively regulated by the bonded H. This positive correlation of *C*_GB_ and *C*_H_ also demonstrates that most of the hydrogen atoms in the nc-Si:H films are located in the grain boundaries, rather than voids or other quasiordered phases, even though the amorphous part accounts for most of the thin films. Grain boundaries can probably offer location for most of the oxygen impurities out of post-oxidization, where the oxygen atoms can incorporate the dangling bonds along the grain boundaries. On the other hand, the incorporation of oxygen impurities in the films is effectively influenced by H radicals. The mechanism is that H radicals generated in the plasma during the growth process of the films are accelerated by the RF power and impinge onto the growing surface of the films with a certain kinetic energy. Those H radicals with enough kinetic energy can passivate the dangling bonds along the grain boundaries and effectively prevent the oxygen impurities from post-oxidization.

The bonded H located at grain boundaries can form hydrides with a certain type of bonding configuration, which can be identified from the deconvoluted peaks of the Si-H stretching mode of the peak at 2,090 cm^-1^ as mentioned in Figure 2a. These hydrides with different types of bonding configurations were then investigated in this part to help us accurately understand the spatial correlation between the hydrogen-related microstructures and oxygen impurities.

**Figure 5:**
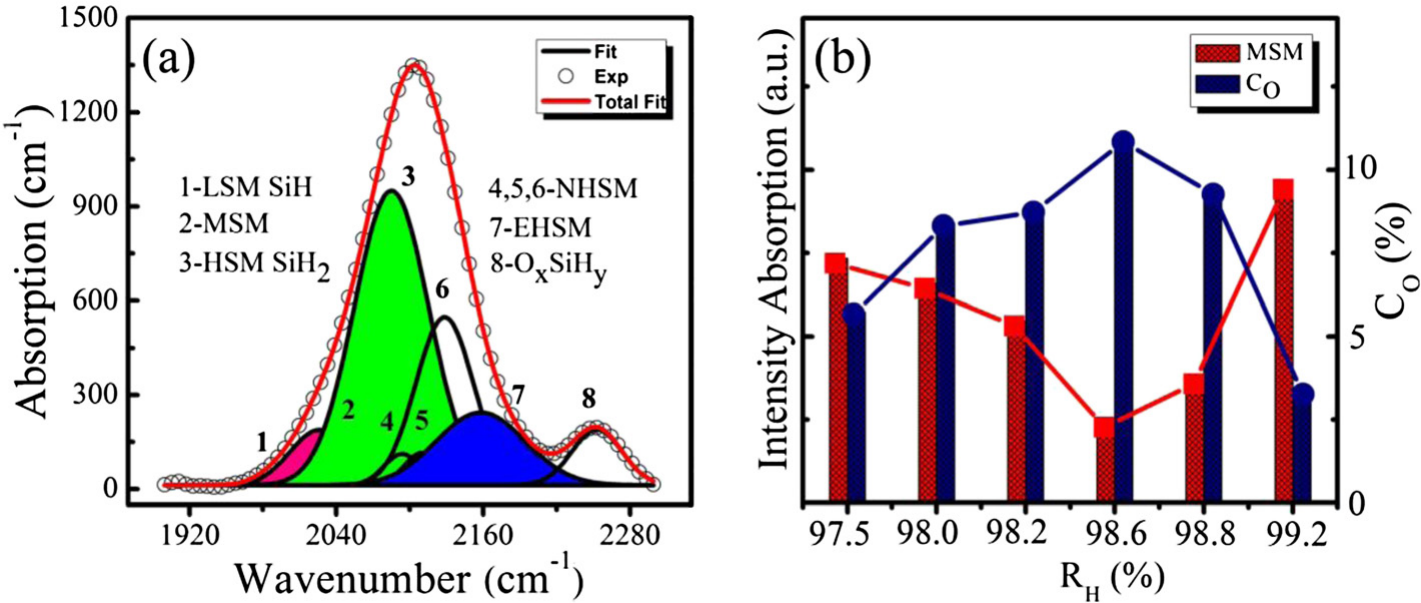
**Deconvoluted Si-H stretching mode and correlation between the integrated intensity of MSM and oxygen content. (a)** Typical deconvoluted Si-H stretching mode of the nc-Si:H thin film under *R*_H_ = 98.2%. The solid curves are the overall fitting results using eight Gaussian peaks. **(b)** Correlation between the integrated intensity of the MSM and the oxygen content as a function of *R*_H_.

The spectrum of a representative sample with *R*_H_ = 98.2% was chosen to be deconvoluted into eight Gaussian absorption peaks as presented in Figure 5a, standing for several types of different bonding configurations. The frequency position of the deconvoluted peaks depends on the unscreened eigen-frequency of the hydride, bulk screening, local hydride density, and possible mutual dipole interactions of the hydrogen incorporation configuration [31]. The low stretching mode (LSM; 1,980 to 2,010 cm^-1^) originating from the a-Si:H tissue is often in an isolated Si-H form in the bulk. The middle stretching mode (MSM; 2,024 to 2,041 cm^-1^) due to the Si-H stretching vibrations is located at the platelet-like configuration of the amorphous-crystalline interface with massive defect states. The high stretching mode (HSM; 2,086 to 2,094 cm^-1^) responsible for Si-H_2_ at the internal surface of microvoids [32] is also related to a number of unsaturated dangling bonds. The extreme HSM (EHSM; 2,140 to 2,150 cm^-1^) arises from the trihydrides in the film prepared under high hydrogen dilution conditions. Three narrow HSMs (NHSMs; at 2,097, 2,109, and 2,137 cm^-1^) represent mono-, di-, and trihydrides, respectively, on the crystalline surface. Lastly, the stretching mode at approximately 2,250 cm^-1^ corresponds to the hydride O_*x*_Si-H_*y*_ vibration with oxygen atoms back-bonded to the silicon atoms [33].

Among the eight bonding configurations of hydrides, the MSM corresponding to the bonding configuration of the hydrides in the grain boundaries is the major mode that determines the mechanism of hydrogen's influence on oxygen impurities. We show in Figure 5b the integrated intensity of the MSM and the bonded oxygen content *C*_O_ for all the samples with *R*_H_ = 97.5% to 99.2%. It is clear that the integrated intensity of the MSM decreases with *R*_H_ increasing from 97.5% to 98.6% and then increases when further increasing *R*_H_ from 98.6% to 99.2%. As also shown in Figure 5b, *C*_O_ has an inverse evolution compared with the integrated intensity of the MSM, illustrating that the MSM is closely related to the oxygen impurities. H atoms and ions incorporate the silicon dangling bonds along the platelet-like configuration of the amorphous-crystalline interface, that is, grain boundaries, and form the hydride corresponding to the MSM. These hydrides located in grain boundaries can effectively passivate the nc-Si:H films by preventing the oxygen incursions from inducing the increase of dangling bonds (P_b_ center defects) [10]. And this inverse correlation between the integrated intensity of the MSM and *C*_O_ further proves that the oxygen impurities mainly reside at the grain boundaries of the nc-Si:H films.

Based on the above results and analysis, we can hereby draw a clear physical picture of the structure evolution mechanism and the effect of the hydrogen behavior on the structure as well as the oxygen impurities in the growth process of the nc-Si:H thin film. The growth of the nc-Si:H thin film is the overall effect of two competing processes: the formation of radicals and the etching of deposition. These two processes are significantly influenced by the proportions of the impinging SiH_*x*_ radicals and atomic hydrogen ions, which vary with different hydrogen dilutions. During the initial stage, increasing *R*_H_ from 97.5% to 98.6% led to the decrease of the density of the SiH_*x*_ radicals, which together with the H etching effect resulted in the decrease of the growth rate. Considering the high RF power density applied on the depositing substrate, the ion bombardment effect [19] should be taken into account. The ion bombardment effect of the increasing H species on the SiH_*x*_ radicals during the growth process reduced the surface diffusion length of film precursors, and these precursors could not reach their favorable growing sites, leading to the formation of more microvoids with amorphous components in the nc-Si:H film. These subsequently formed microvoids induced larger areas of internal surfaces with dangling bonds and weaker Si-Si bonds in the growing film. Therefore, through H ion implantation, atomic hydrogen diffusion, and relevant chemical reactions, more hydrogen would be bonded to silicon or trapped in these microvoids, resulting in not only an increase in the volume fraction of voids *P*_V_ within the film as shown in Figure 2b but also a decrease in both the grain size *d* and the crystalline volume fraction *X*_C_, as observed in both the XRD and Raman measurements presented in Figure 1a,c. In the meanwhile, the enhanced H abstraction reaction [34,35] of the increasing H atoms and ions took away a certain number of the bonded H from the hydrides at grain boundaries, and more oxygen impurities could incorporate the dangling bonds at grain boundaries, giving rise to the decrease of the integrated intensity of the MSM and the increase of *C*_O_ as shown in Figure 5b.

Further increasing *R*_H_ from 98.6% to 99.2% led to a declining growth rate due to the further decreasing density of the SiH_*x*_ radicals. At the same time, the *P*_V_ of the growing film was further enhanced (see Figure 2b) because of the ion bombardment effect of the excessive H species. However, in this *R*_H_ range, 98.6% to 99.2%, the hydrogen-induced annealing effect [36] gradually became dominant over the effect of the ion bombardment-induced amorphization. The excessive H species presenting on the growing surface of the film could penetrate into the subsurface and rearrange the Si-Si network structure. These H atoms and ions saturated the present dangling bonds at the interface between the amorphous and crystalline regions and formed molecular hydrogen through the reaction of adsorbed hydrogen with clustered hydrogen in the subsurface, which was less mobile than the atomic hydrogen. Further H insertion reaction with the a-Si:H matrix destructed and perturbed the strained Si-Si bonds, and the subsequent structural relaxation of the Si-Si bonds resulted in the transformation of the film's structure from amorphous to nanocrystalline. Therefore, as a general result, excessive hydrogen presenting in the plasma could lead to a greater probability of crystallization, supported by the observation of *X*_C_ in Figure 1c. The slight enhancement of the grain size *d* from 5.5 to 6.1 nm as seen in Figure 1a without any remarkable change can be attributed to the suppression of the growth by the excessive H ion implantation on the nucleation site, as well as the depletion of the SiH_*x*_ radical by the hydrogen flux. On the other hand, the results of the increasing integrated intensity of the MSM and the decreasing *C*_O_ as shown in Figure 5b in this *R*_H_ range illustrate that those H atoms and ions penetrating into the subsurface could saturate the dangling bonds along the grain boundaries, and more hydrides were formed to effectively avoid the post-oxidation effect by preventing the oxygen impurities from incorporating the dangling bonds in the grain boundaries. Hence, compact-structure and well-passivated grain boundaries are less susceptible to oxygen impurities. Our previous work of applying an extra negative bias on the substrate [37] offers an effective way to lower the defect density and the oxygen impurities inside nc-Si:H films.

## Conclusions

In summary, we have conducted a detailed investigation on the mechanism of hydrogen's influence on structure evolution and oxygen impurities from a series of nc-Si:H thin films prepared under different hydrogen dilution ratio treatment in PECVD. XRD, TEM, Raman, and optical transmission techniques have been utilized to understand the microstructure characterization of nc-Si:H thin films. XPS results have confirmed that oxygen impurities on the surface of the nc-Si:H films have the dominant formation state of SiO_2_. The good agreement between the bonded hydrogen content and the volume fraction of grain boundary illustrates that as an important defect structure, the volume fraction of grain boundary in nc-Si:H films can be effectively regulated through hydrogen dilution. The inverse relationship between the integrated intensity of MSM and the oxygen content presents that the oxygen incursions due to post-oxidation originate from the location of grain boundaries inside nc-Si:H films. The tuning mechanism of hydrogen on oxygen impurities is that the hydrides corresponding to the MSM with a certain kind of bonding configuration are formed by the incorporation of H atoms and ions with the silicon dangling bonds located at grain boundaries, which can effectively prevent the oxygen incursions from residing along grain boundaries and further forming the Si-O/Si defects. Therefore, applying an extra negative bias on the substrate during the growth process is proposed to reduce the probability of oxygen contamination, which can produce films with better light absorption properties in the solar cell application.

## Competing interests

The authors declare that they have no competing interests.

## Authors' contributions

CW participated in the design of the study, carried out the experiments, and performed the statistical analysis, as well as drafted the manuscript. HX, WH, and ZPL participated in the design of the study and provided the experimental guidance. WZS designed the study, took charge of the overall guidance, and revised the manuscript. All authors read and approved the final manuscript.
